# Editorial: Immuno-metabolic approaches for the treatment of hepatobiliary and pancreatic tumors

**DOI:** 10.3389/fimmu.2026.1810064

**Published:** 2026-02-23

**Authors:** Yuanxia Han, Luyu Yang, Jianwei Xu, Hengrui Liu, Ran Wei

**Affiliations:** 1 State Key Laboratory of Oncology in South China, Guangdong Provincial Clinical Research Center for Cancer, Sun Yat‐sen University Cancer Center, Guangzhou, Guangdong, China; 2 Hepatobiliary Surgery, Department of General Surgery, Huashan Hospital, Fudan University, Shanghai, China; 3 Department of Surgery, Qilu Hospital, Shandong University, Jinan, China; 4 Department of Biochemistry, University of Cambridge, Cambridge, United Kingdom

**Keywords:** combination therapy, hepatobiliary tumors, immuno-metabolic approach, pancreatic cancer, tumor microenvironment (TME)

The field of hepatobiliary and pancreatic tumor therapy has witnessed significant advancements in recent years, particularly with the integration of immuno-metabolic approaches. This Research Topic, titled *“Immuno-metabolic Approaches for the Treatment of Hepatobiliary and Pancreatic Tumors,”* has garnered substantial attention, featuring a Research Topic of insightful studies that delve into the intricate interplay between immune responses and metabolic pathways in these challenging malignancies.

## Immuno-metabolic dynamics in pancreatic tumors

The work by Hui et al. provides a comprehensive overview of the immunosuppressive tumor microenvironment (TME) in pancreatic ductal adenocarcinoma (PDAC). Their study elucidates how dense extracellular matrix, cancer-associated fibroblasts (CAFs), and myeloid-derived suppressor cells (MDSCs) contribute to immune evasion. By focusing on the metabolic reprogramming of PDAC, they highlight potential strategies to overcome immunotherapy resistance, emphasizing the importance of targeting both immune and metabolic checkpoints. Similarly, Qin et al. have developed a nomogram that integrates immune checkpoints, fibrosis indicators, and clinicopathological characteristics to predict overall survival in pancreatic cancer. This study underscores the significance of considering both immune and metabolic factors in prognostic assessments, offering clinicians a valuable tool for personalized treatment planning. These studies collectively underscore the complexity of the PDAC TME and the need for multifaceted therapeutic interventions. By elucidating the interplay between immune evasion and metabolic reprogramming, they pave the way for innovative combination therapies that can overcome current treatment limitations. Recent research continues to explore the potential of targeting the TME to improve immunotherapy outcomes. For instance, strategies aimed at disrupting the metabolic symbiosis between tumor cells and stromal components or reprogramming immunosuppressive cells are gaining traction (1, 2). As the field progresses, integrating immune-metabolic approaches into clinical practice holds great promise for enhancing the survival and quality of life of PDAC patients.

## Hepatocellular carcinoma: bridging immunity and metabolism

In hepatocellular carcinoma (HCC), the interplay between immunity and metabolism is equally critical. The study by Lian et al. explores the role of neutrophils in the HCC microenvironment, revealing their dual role in promoting and suppressing tumor immunity. Neutrophils, through the release of reactive oxygen species and various cytokines, can both facilitate tumor progression and enhance anti-tumor immune responses. This duality highlights the complexity of the HCC TME and the need for targeted interventions that can harness the anti-tumor potential of neutrophils while mitigating their pro-tumor effects. Moreover, Xing et al. have identified serum GDF15 levels as a predictive biomarker for clinical outcomes in patients with unresectable HCC treated with hepatic arterial infusion chemotherapy (HAIC). Their findings suggest that GDF15, a metabolic regulator, plays a crucial role in modulating immune responses and could serve as a therapeutic target to enhance the efficacy of HAIC.

## Combination therapies and prognostic models

The efficacy of combination therapies is another focal point of this Research Topic. The systematic review by Ye et al. demonstrates that the combination of transarterial chemoembolization (TACE), tyrosine kinase inhibitors (TKIs), and immune checkpoint inhibitors (ICIs) significantly improves outcomes in patients with unresectable HCC. Similarly, Cao et al. showed that HAIC plus PD-1 inhibitors and TKIs prolonged survival in HCC with PVTT, particularly in younger patients. Furthermore, the work by Luo et al. and their colleagues comprehensively evaluated the efficacy of combining HAIC with molecular targeted therapies and PD-(L)1 inhibitors (HAIT-M-P) versus HAIC alone in patients with locally aggressive early recurrent hepatocellular carcinoma (erHCC), suggesting HAIT-M-P as a superior treatment strategy for enhancing outcomes in erHCC. Their findings support the use of such combinations in clinical practice, emphasizing the need for further research to refine patient selection and treatment protocols.

## Future directions and challenges

Looking ahead, the integration of immuno - metabolic approaches in hepatobiliary and pancreatic tumor therapy stands as a beacon of hope, brimming with immense potential. The intricate relationship between the immune system and metabolic processes within the tumor microenvironment (TME) has been increasingly recognized as a key determinant of tumor progression and treatment response (3).

However, numerous challenges loom on the horizon. Identifying reliable biomarkers for treatment response remains an arduous task. These biomarkers are crucial for early prediction of therapy efficacy, enabling timely adjustments to treatment plans (4). The development of effective combination therapies is another significant hurdle. While current studies have shown promise in combining different treatment modalities, optimizing the sequence and dosage of these combinations to maximize efficacy and minimize side effects is still a work in progress. Moreover, optimizing patient selection criteria is essential to ensure that the right patients receive the most appropriate treatments. Future research should prioritize elucidating the underlying mechanisms of immune - metabolic interactions. By understanding how metabolic changes influence immune cell function and vice versa, we can identify novel therapeutic targets. Well - designed clinical trials are then imperative to validate the efficacy of these new approaches.

In conclusion, the studies in this Research Topic offer invaluable insights into the complex interplay between immunity and metabolism in hepatobiliary and pancreatic tumors. As experts in the field, we must harness these insights to develop more effective and personalized treatment strategies, ultimately improving patient outcomes and driving the progress of oncology. The integration of immuno - metabolic approaches will undoubtedly be a cornerstone in the future of cancer therapy.
